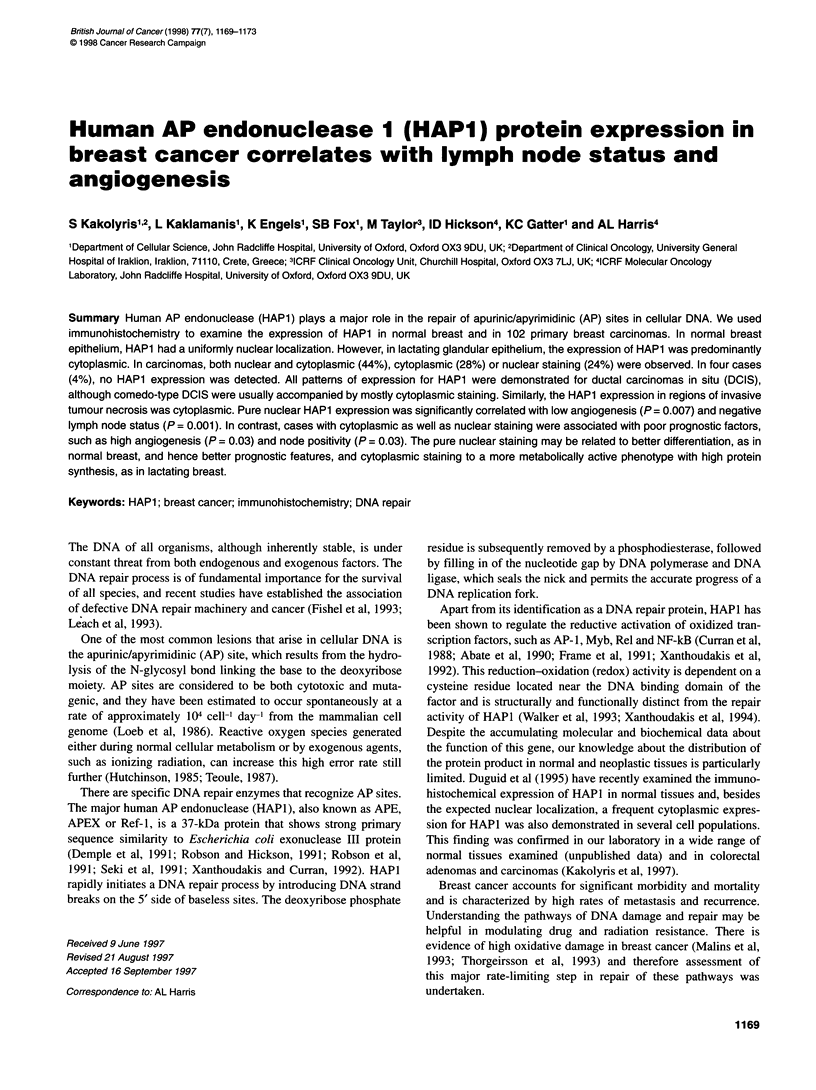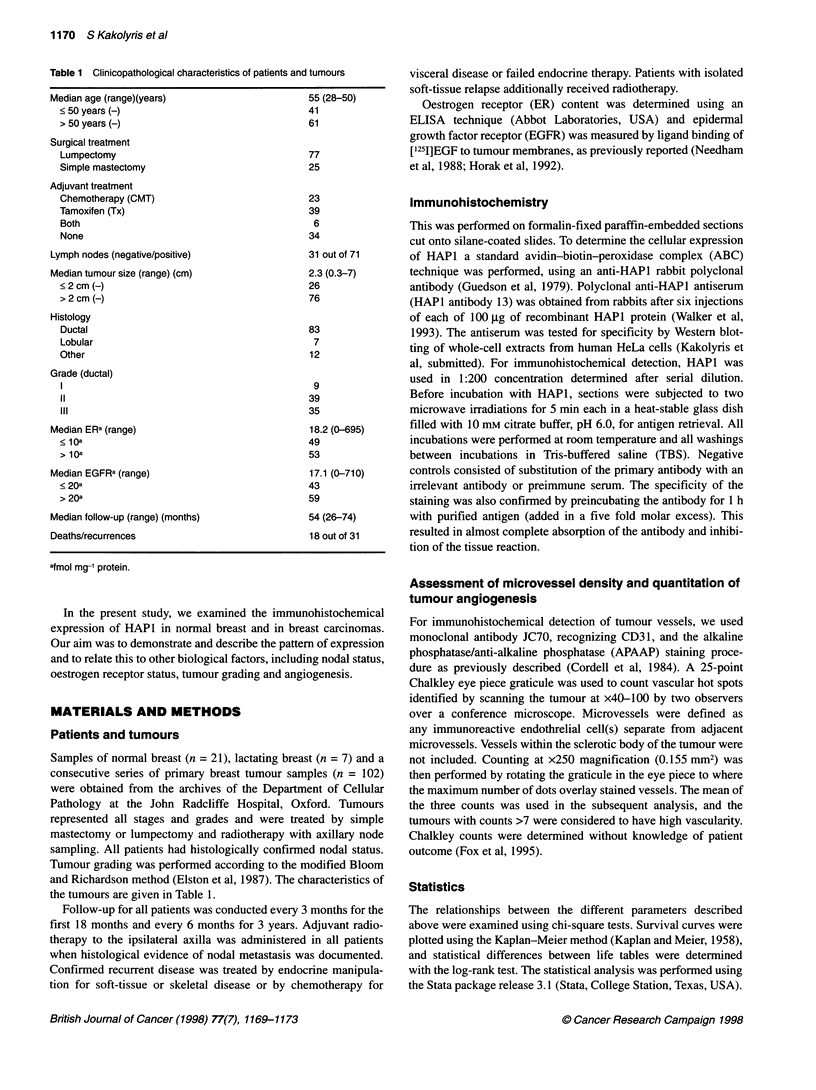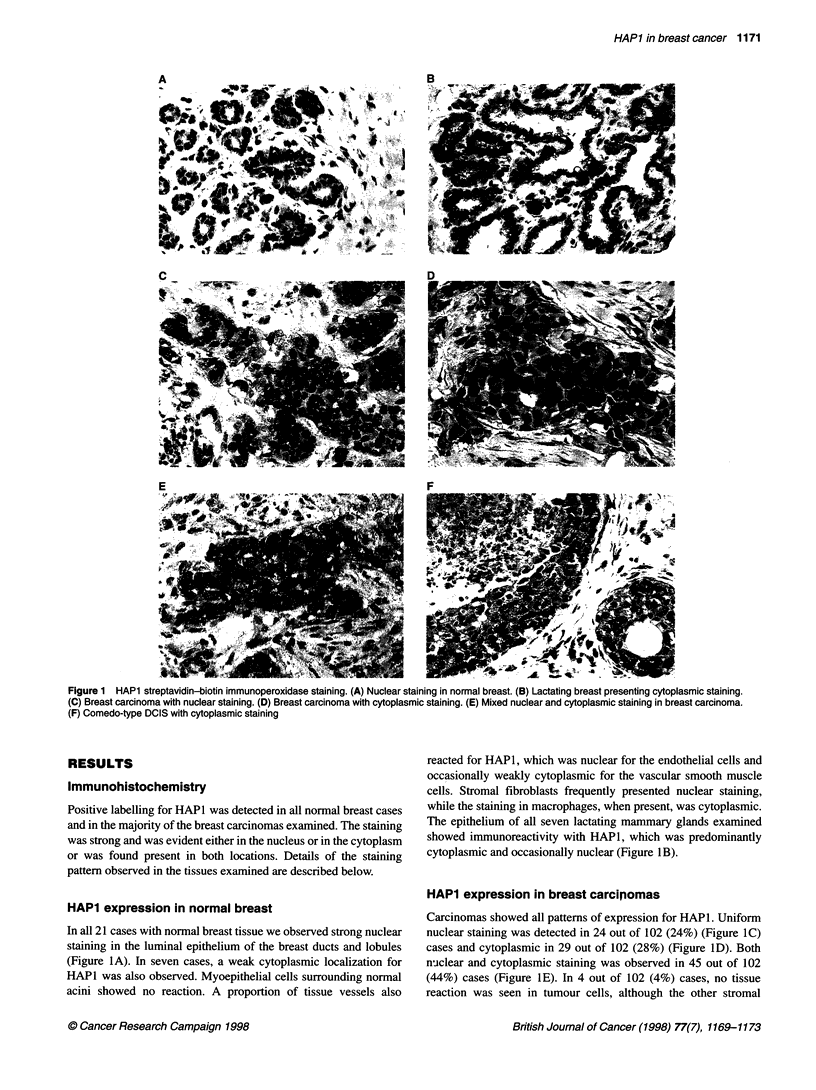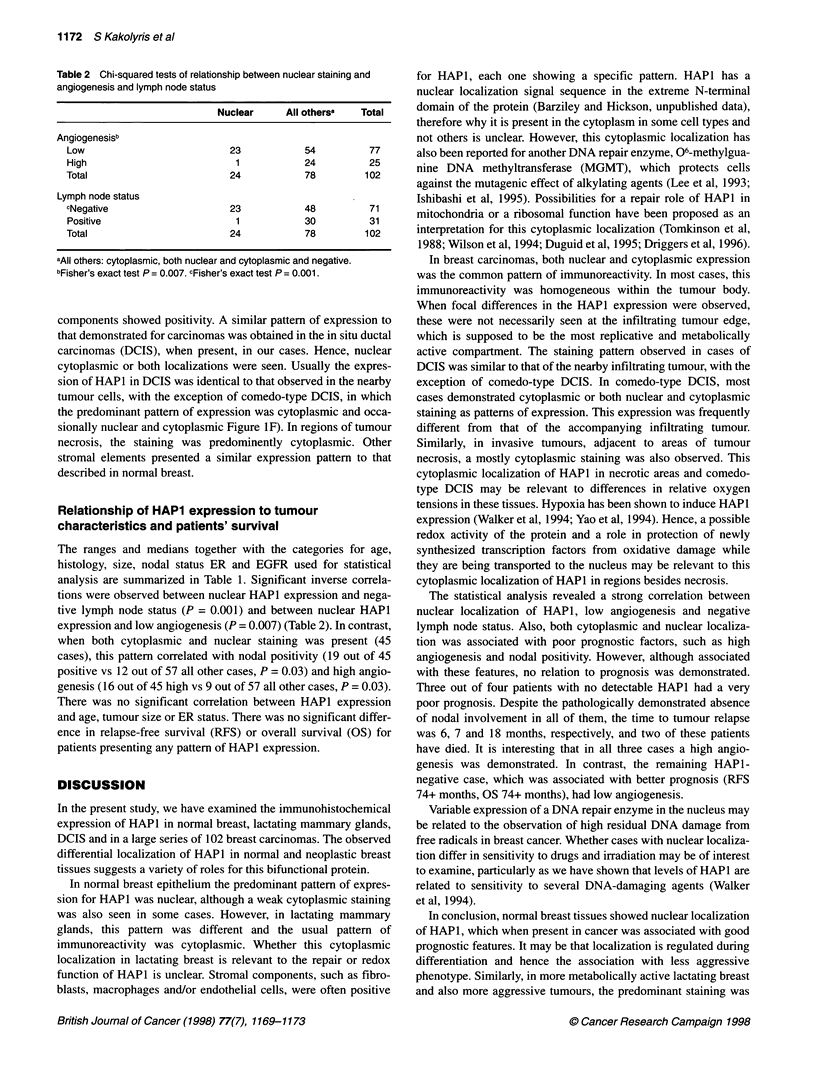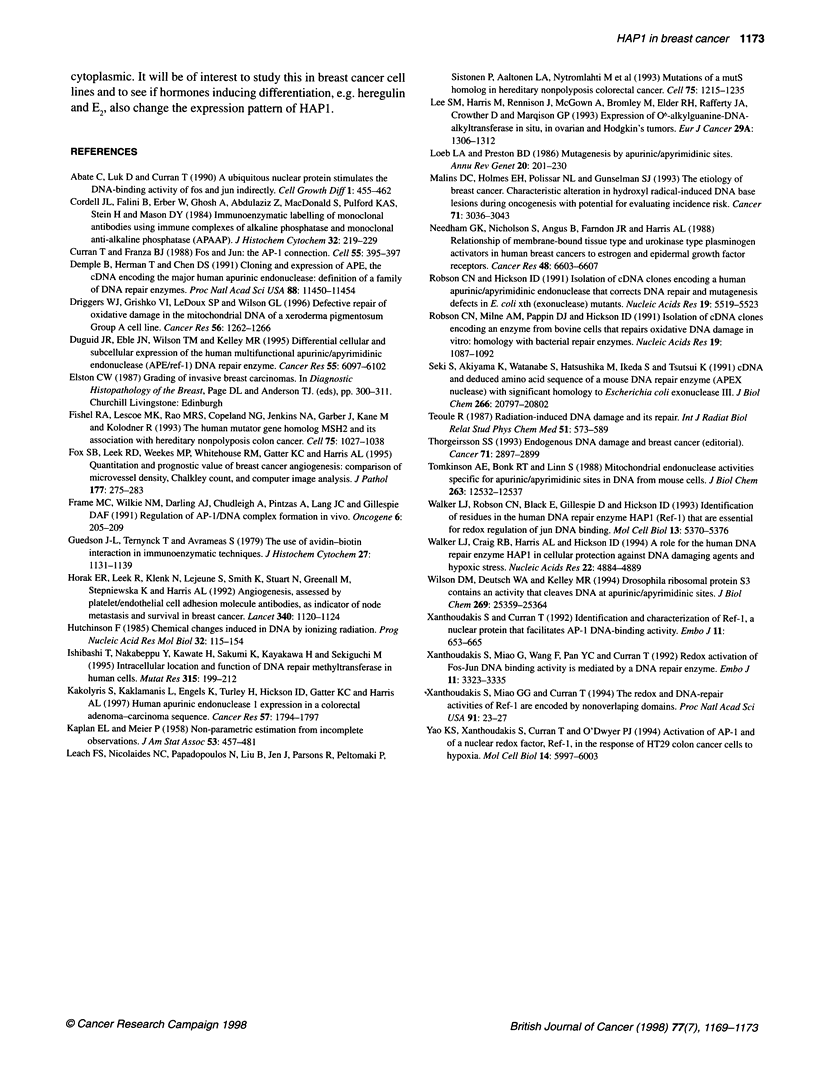# Human AP endonuclease 1 (HAP1) protein expression in breast cancer correlates with lymph node status and angiogenesis.

**DOI:** 10.1038/bjc.1998.194

**Published:** 1998-04

**Authors:** S. Kakolyris, L. Kaklamanis, K. Engels, S. B. Fox, M. Taylor, I. D. Hickson, K. C. Gatter, A. L. Harris

**Affiliations:** Department of Cellular Science, John Radcliffe Hospital, University of Oxford, UK.

## Abstract

**Images:**


British Journal of Cancer (1998) 77(7), 1169–1173
© 1998 Cancer Research Campaign

### Human AP endonuclease 1 (HAP1) protein expression in breast cancer correlates with lymph node status and angiogenesis

**S Kakolyris[1,2], L Kaklamanis[1], K Engels[1], SB Fox[1], M Taylor[3], ID Hickson[4], KC Gatter[1] and AL Harris[4]**

[1]Department of Cellular Science, John Radcliffe Hospital, University of Oxford, Oxford OX3 9DU, UK; [2]Department of Clinical Oncology, University General Hospital of Iraklion, Iraklion, 71110, Crete, Greece; [3]ICRF Clinical Oncology Unit, Churchill Hospital, Oxford OX3 7LJ, UK; [4]ICRF Molecular Oncology Laboratory, John Radcliffe Hospital, University of Oxford, Oxford OX3 9DU, UK

**Summary** Human AP endonuclease (HAP1) plays a major role in the repair of apurinic/apyrimidinic (AP) sites in cellular DNA. We used immunohistochemistry to examine the expression of HAP1 in normal breast and in 102 primary breast carcinomas. In normal breast epithelium, HAP1 had a uniformly nuclear localization. However, in lactating glandular epithelium, the expression of HAP1 was predominantly cytoplasmic. In carcinomas, both nuclear and cytoplasmic (44%), cytoplasmic (28%) or nuclear staining (24%) were observed. In four cases (4%), no HAP1 expression was detected. All patterns of expression for HAP1 were demonstrated for ductal carcinomas in situ (DCIS), although comedo-type DCIS were usually accompanied by mostly cytoplasmic staining. Similarly, the HAP1 expression in regions of invasive tumour necrosis was cytoplasmic. Pure nuclear HAP1 expression was significantly correlated with low angiogenesis ($P = 0.007$) and negative lymph node status ($P = 0.001$). In contrast, cases with cytoplasmic as well as nuclear staining were associated with poor prognostic factors, such as high angiogenesis ($P = 0.03$) and node positivity ($P = 0.03$). The pure nuclear staining may be related to better differentiation, as in normal breast, and hence better prognostic features, and cytoplasmic staining to a more metabolically active phenotype with high protein synthesis, as in lactating breast.

**Keywords:** HAP1; breast cancer; immunohistochemistry; DNA repair

The DNA of all organisms, although inherently stable, is under constant threat from both endogenous and exogenous factors. The DNA repair process is of fundamental importance for the survival of all species, and recent studies have established the association of defective DNA repair machinery and cancer (Fishel et al, 1993; Leach et al, 1993).

One of the most common lesions that arise in cellular DNA is the apurinic/apyrimidinic (AP) site, which results from the hydrolysis of the N-glycosyl bond linking the base to the deoxyribose moiety. AP sites are considered to be both cytotoxic and mutagenic, and they have been estimated to occur spontaneously at a rate of approximately $10^4$ cell$^{-1}$ day$^{-1}$ from the mammalian cell genome (Loeb et al, 1986). Reactive oxygen species generated either during normal cellular metabolism or by exogenous agents, such as ionizing radiation, can increase this high error rate still further (Hutchinson, 1985; Teoule, 1987).

There are specific DNA repair enzymes that recognize AP sites. The major human AP endonuclease (HAP1), also known as APE, APEX or Ref-1, is a 37-kDa protein that shows strong primary sequence similarity to *Escherichia coli* exonuclease III protein (Demple et al, 1991; Robson and Hickson, 1991; Robson et al, 1991; Seki et al, 1991; Xanthoudakis and Curran, 1992). HAP1 rapidly initiates a DNA repair process by introducing DNA strand breaks on the 5′ side of baseless sites. The deoxyribose phosphate residue is subsequently removed by a phosphodiesterase, followed by filling in of the nucleotide gap by DNA polymerase and DNA ligase, which seals the nick and permits the accurate progress of a DNA replication fork.

*Received 9 June 1997*
*Revised 21 August 1997*
*Accepted 16 September 1997*

*Correspondence to:* AL Harris

Apart from its identification as a DNA repair protein, HAP1 has been shown to regulate the reductive activation of oxidized transcription factors, such as AP-1, Myb, Rel and NF-kB (Curran et al, 1988; Abate et al, 1990; Frame et al, 1991; Xanthoudakis et al, 1992). This reduction–oxidation (redox) activity is dependent on a cysteine residue located near the DNA binding domain of the factor and is structurally and functionally distinct from the repair activity of HAP1 (Walker et al, 1993; Xanthoudakis et al, 1994). Despite the accumulating molecular and biochemical data about the function of this gene, our knowledge about the distribution of the protein product in normal and neoplastic tissues is particularly limited. Duguid et al (1995) have recently examined the immunohistochemical expression of HAP1 in normal tissues and, besides the expected nuclear localization, a frequent cytoplasmic expression for HAP1 was also demonstrated in several cell populations. This finding was confirmed in our laboratory in a wide range of normal tissues examined (unpublished data) and in colorectal adenomas and carcinomas (Kakolyris et al, 1997).

Breast cancer accounts for significant morbidity and mortality and is characterized by high rates of metastasis and recurrence. Understanding the pathways of DNA damage and repair may be helpful in modulating drug and radiation resistance. There is evidence of high oxidative damage in breast cancer (Malins et al, 1993; Thorgeirsson et al, 1993) and therefore assessment of this major rate-limiting step in repair of these pathways was undertaken.

**Table 1** Clinicopathological characteristics of patients and tumours

| | |
|---|---|
| Median age (range)(years) | 55 (28–50) |
| ≤ 50 years (–) | 41 |
| > 50 years (–) | 61 |
| Surgical treatment | |
| Lumpectomy | 77 |
| Simple mastectomy | 25 |
| Adjuvant treatment | |
| Chemotherapy (CMT) | 23 |
| Tamoxifen (Tx) | 39 |
| Both | 6 |
| None | 34 |
| Lymph nodes (negative/positive) | 31 out of 71 |
| Median tumour size (range) (cm) | 2.3 (0.3–7) |
| ≤ 2 cm (–) | 26 |
| > 2 cm (–) | 76 |
| Histology | |
| Ductal | 83 |
| Lobular | 7 |
| Other | 12 |
| Grade (ductal) | |
| I | 9 |
| II | 39 |
| III | 35 |
| Median ER[a] (range) | 18.2 (0–695) |
| ≤ 10[a] | 49 |
| > 10[a] | 53 |
| Median EGFR[a] (range) | 17.1 (0–710) |
| ≤ 20[a] | 43 |
| > 20[a] | 59 |
| Median follow-up (range) (months) | 54 (26–74) |
| Deaths/recurrences | 18 out of 31 |

[a]fmol $mg^{-1}$ protein.

In the present study, we examined the immunohistochemical expression of HAP1 in normal breast and in breast carcinomas. Our aim was to demonstrate and describe the pattern of expression and to relate this to other biological factors, including nodal status, oestrogen receptor status, tumour grading and angiogenesis.

### MATERIALS AND METHODS

### Patients and tumours

Samples of normal breast ($n$ = 21), lactating breast ($n$ = 7) and a consecutive series of primary breast tumour samples ($n$ = 102) were obtained from the archives of the Department of Cellular Pathology at the John Radcliffe Hospital, Oxford. Tumours represented all stages and grades and were treated by simple mastectomy or lumpectomy and radiotherapy with axillary node sampling. All patients had histologically confirmed nodal status. Tumour grading was performed according to the modified Bloom and Richardson method (Elston et al, 1987). The characteristics of the tumours are given in Table 1.

Follow-up for all patients was conducted every 3 months for the first 18 months and every 6 months for 3 years. Adjuvant radiotherapy to the ipsilateral axilla was administered in all patients when histological evidence of nodal metastasis was documented. Confirmed recurrent disease was treated by endocrine manipulation for soft-tissue or skeletal disease or by chemotherapy for visceral disease or failed endocrine therapy. Patients with isolated soft-tissue relapse additionally received radiotherapy.

Oestrogen receptor (ER) content was determined using an ELISA technique (Abbot Laboratories, USA) and epidermal growth factor receptor (EGFR) was measured by ligand binding of [$^{125}$I]EGF to tumour membranes, as previously reported (Needham et al, 1988; Horak et al, 1992).

### Immunohistochemistry

This was performed on formalin-fixed paraffin-embedded sections cut onto silane-coated slides. To determine the cellular expression of HAP1 a standard avidin–biotin–peroxidase complex (ABC) technique was performed, using an anti-HAP1 rabbit polyclonal antibody (Guedson et al, 1979). Polyclonal anti-HAP1 antiserum (HAP1 antibody 13) was obtained from rabbits after six injections of each of 100 µg of recombinant HAP1 protein (Walker et al, 1993). The antiserum was tested for specificity by Western blotting of whole-cell extracts from human HeLa cells (Kakolyris et al, submitted). For immunohistochemical detection, HAP1 was used in 1:200 concentration determined after serial dilution. Before incubation with HAP1, sections were subjected to two microwave irradiations for 5 min each in a heat-stable glass dish filled with 10 mM citrate buffer, pH 6.0, for antigen retrieval. All incubations were performed at room temperature and all washings between incubations in Tris-buffered saline (TBS). Negative controls consisted of substitution of the primary antibody with an irrelevant antibody or preimmune serum. The specificity of the staining was also confirmed by preincubating the antibody for 1 h with purified antigen (added in a five fold molar excess). This resulted in almost complete absorption of the antibody and inhibition of the tissue reaction.

### Assessment of microvessel density and quantitation of tumour angiogenesis

For immunohistochemical detection of tumour vessels, we used monoclonal antibody JC70, recognizing CD31, and the alkaline phosphatase/anti-alkaline phosphatase (APAAP) staining procedure as previously described (Cordell et al, 1984). A 25-point Chalkley eye piece graticule was used to count vascular hot spots identified by scanning the tumour at ×40–100 by two observers over a conference microscope. Microvessels were defined as any immunoreactive endothrelial cell(s) separate from adjacent microvessels. Vessels within the sclerotic body of the tumour were not included. Counting at ×250 magnification (0.155 $mm^2$) was then performed by rotating the graticule in the eye piece to where the maximum number of dots overlay stained vessels. The mean of the three counts was used in the subsequent analysis, and the tumours with counts >7 were considered to have high vascularity. Chalkley counts were determined without knowledge of patient outcome (Fox et al, 1995).

### Statistics

The relationships between the different parameters described above were examined using chi-square tests. Survival curves were plotted using the Kaplan–Meier method (Kaplan and Meier, 1958), and statistical differences between life tables were determined with the log-rank test. The statistical analysis was performed using the Stata package release 3.1 (Stata, College Station, Texas, USA).

© Cancer Research Campaign 1998

**Figure 1** HAP1 streptavidin–biotin immunoperoxidase staining. (**A**) Nuclear staining in normal breast. (**B**) Lactating breast presenting cytoplasmic staining. (**C**) Breast carcinoma with nuclear staining. (**D**) Breast carcinoma with cytoplasmic staining. (**E**) Mixed nuclear and cytoplasmic staining in breast carcinoma. (**F**) Comedo-type DCIS with cytoplasmic staining

### RESULTS

### Immunohistochemistry

Positive labelling for HAP1 was detected in all normal breast cases and in the majority of the breast carcinomas examined. The staining was strong and was evident either in the nucleus or in the cytoplasm or was found present in both locations. Details of the staining pattern observed in the tissues examined are described below.

### HAP1 expression in normal breast

In all 21 cases with normal breast tissue we observed strong nuclear staining in the luminal epithelium of the breast ducts and lobules (Figure 1A). In seven cases, a weak cytoplasmic localization for HAP1 was also observed. Myoepithelial cells surrounding normal acini showed no reaction. A proportion of tissue vessels also reacted for HAP1, which was nuclear for the endothelial cells and occasionally weakly cytoplasmic for the vascular smooth muscle cells. Stromal fibroblasts frequently presented nuclear staining, while the staining in macrophages, when present, was cytoplasmic. The epithelium of all seven lactating mammary glands examined showed immunoreactivity with HAP1, which was predominantly cytoplasmic and occasionally nuclear (Figure 1B).

### HAP1 expression in breast carcinomas

Carcinomas showed all patterns of expression for HAP1. Uniform nuclear staining was detected in 24 out of 102 (24%) (Figure 1C) cases and cytoplasmic in 29 out of 102 (28%) (Figure 1D). Both nuclear and cytoplasmic staining was observed in 45 out of 102 (44%) cases (Figure 1E). In 4 out of 102 (4%) cases, no tissue reaction was seen in tumour cells, although the other stromal

© Cancer Research Campaign 1998

**Table 2** Chi-squared tests of relationship between nuclear staining and angiogenesis and lymph node status

| | Nuclear | All others[a] | Total |
|---|---|---|---|
| Angiogenesis[b] | | | |
| Low | 23 | 54 | 77 |
| High | 1 | 24 | 25 |
| Total | 24 | 78 | 102 |
| Lymph node status | | | |
| [c]Negative | 23 | 48 | 71 |
| Positive | 1 | 30 | 31 |
| Total | 24 | 78 | 102 |

[a]All others: cytoplasmic, both nuclear and cytoplasmic and negative.
[b]Fisher's exact test $P = 0.007$. [c]Fisher's exact test $P = 0.001$.

components showed positivity. A similar pattern of expression to that demonstrated for carcinomas was obtained in the in situ ductal carcinomas (DCIS), when present, in our cases. Hence, nuclear cytoplasmic or both localizations were seen. Usually the expression of HAP1 in DCIS was identical to that observed in the nearby tumour cells, with the exception of comedo-type DCIS, in which the predominant pattern of expression was cytoplasmic and occasionally nuclear and cytoplasmic Figure 1F). In regions of tumour necrosis, the staining was predominently cytoplasmic. Other stromal elements presented a similar expression pattern to that described in normal breast.

### Relationship of HAP1 expression to tumour characteristics and patients' survival

The ranges and medians together with the categories for age, histology, size, nodal status ER and EGFR used for statistical analysis are summarized in Table 1. Significant inverse correlations were observed between nuclear HAP1 expression and negative lymph node status ($P = 0.001$) and between nuclear HAP1 expression and low angiogenesis ($P = 0.007$) (Table 2). In contrast, when both cytoplasmic and nuclear staining was present (45 cases), this pattern correlated with nodal positivity (19 out of 45 positive vs 12 out of 57 all other cases, $P = 0.03$) and high angiogenesis (16 out of 45 high vs 9 out of 57 all other cases, $P = 0.03$). There was no significant correlation between HAP1 expression and age, tumour size or ER status. There was no significant difference in relapse-free survival (RFS) or overall survival (OS) for patients presenting any pattern of HAP1 expression.

### DISCUSSION

In the present study, we have examined the immunohistochemical expression of HAP1 in normal breast, lactating mammary glands, DCIS and in a large series of 102 breast carcinomas. The observed differential localization of HAP1 in normal and neoplastic breast tissues suggests a variety of roles for this bifunctional protein.

In normal breast epithelium the predominant pattern of expression for HAP1 was nuclear, although a weak cytoplasmic staining was also seen in some cases. However, in lactating mammary glands, this pattern was different and the usual pattern of immunoreactivity was cytoplasmic. Whether this cytoplasmic localization in lactating breast is relevant to the repair or redox function of HAP1 is unclear. Stromal components, such as fibroblasts, macrophages and/or endothelial cells, were often positive for HAP1, each one showing a specific pattern. HAP1 has a nuclear localization signal sequence in the extreme N-terminal domain of the protein (Barziley and Hickson, unpublished data), therefore why it is present in the cytoplasm in some cell types and not others is unclear. However, this cytoplasmic localization has also been reported for another DNA repair enzyme, $O^6$-methylguanine DNA methyltransferase (MGMT), which protects cells against the mutagenic effect of alkylating agents (Lee et al, 1993; Ishibashi et al, 1995). Possibilities for a repair role of HAP1 in mitochondria or a ribosomal function have been proposed as an interpretation for this cytoplasmic localization (Tomkinson et al, 1988; Wilson et al, 1994; Duguid et al, 1995; Driggers et al, 1996).

In breast carcinomas, both nuclear and cytoplasmic expression was the common pattern of immunoreactivity. In most cases, this immunoreactivity was homogeneous within the tumour body. When focal differences in the HAP1 expression were observed, these were not necessarily seen at the infiltrating tumour edge, which is supposed to be the most replicative and metabolically active compartment. The staining pattern observed in cases of DCIS was similar to that of the nearby infiltrating tumour, with the exception of comedo-type DCIS. In comedo-type DCIS, most cases demonstrated cytoplasmic or both nuclear and cytoplasmic staining as patterns of expression. This expression was frequently different from that of the accompanying infiltrating tumour. Similarly, in invasive tumours, adjacent to areas of tumour necrosis, a mostly cytoplasmic staining was also observed. This cytoplasmic localization of HAP1 in necrotic areas and comedo-type DCIS may be relevant to differences in relative oxygen tensions in these tissues. Hypoxia has been shown to induce HAP1 expression (Walker et al, 1994; Yao et al, 1994). Hence, a possible redox activity of the protein and a role in protection of newly synthesized transcription factors from oxidative damage while they are being transported to the nucleus may be relevant to this cytoplasmic localization of HAP1 in regions besides necrosis.

The statistical analysis revealed a strong correlation between nuclear localization of HAP1, low angiogenesis and negative lymph node status. Also, both cytoplasmic and nuclear localization was associated with poor prognostic factors, such as high angiogenesis and nodal positivity. However, although associated with these features, no relation to prognosis was demonstrated. Three out of four patients with no detectable HAP1 had a very poor prognosis. Despite the pathologically demonstrated absence of nodal involvement in all of them, the time to tumour relapse was 6, 7 and 18 months, respectively, and two of these patients have died. It is interesting that in all three cases a high angiogenesis was demonstrated. In contrast, the remaining HAP1-negative case, which was associated with better prognosis (RFS 74+ months, OS 74+ months), had low angiogenesis.

Variable expression of a DNA repair enzyme in the nucleus may be related to the observation of high residual DNA damage from free radicals in breast cancer. Whether cases with nuclear localization differ in sensitivity to drugs and irradiation may be of interest to examine, particularly as we have shown that levels of HAP1 are related to sensitivity to several DNA-damaging agents (Walker et al, 1994).

In conclusion, normal breast tissues showed nuclear localization of HAP1, which when present in cancer was associated with good prognostic features. It may be that localization is regulated during differentiation and hence the association with less aggressive phenotype. Similarly, in more metabolically active lactating breast and also more aggressive tumours, the predominant staining was

© Cancer Research Campaign 1998

cytoplasmic. It will be of interest to study this in breast cancer cell lines and to see if hormones inducing differentiation, e.g. heregulin and $E_2$, also change the expression pattern of HAP1.

### REFERENCES

Abate C, Luk D and Curran T (1990) A ubiquitous nuclear protein stimulates the DNA-binding activity of fos and jun indirectly. *Cell Growth Diff* **1**: 455–462

Cordell JL, Falini B, Erber W, Ghosh A, Abdulaziz Z, MacDonald S, Pulford KAS, Stein H and Mason DY (1984) Immunoenzymatic labelling of monoclonal antibodies using immune complexes of alkaline phosphatase and monoclonal anti-alkaline phosphatase (APAAP). *J Histochem Cytochem* **32**: 219–229

Curran T and Franza BJ (1988) Fos and Jun: the AP-1 connection. *Cell* **55**: 395–397

Demple B, Herman T and Chen DS (1991) Cloning and expression of APE, the cDNA encoding the major human apurinic endonuclease: definition of a family of DNA repair enzymes. *Proc Natl Acad Sci USA* **88**: 11450–11454

Driggers WJ, Grishko VI, LeDoux SP and Wilson GL (1996) Defective repair of oxidative damage in the mitochondrial DNA of a xeroderma pigmentosum Group A cell line. *Cancer Res* **56**: 1262–1266

Duguid JR, Eble JN, Wilson TM and Kelley MR (1995) Differential cellular and subcellular expression of the human multifunctional apurinic/apyrimidinic endonuclease (APE/ref-1) DNA repair enzyme. *Cancer Res* **55**: 6097–6102

Elston CW (1987) Grading of invasive breast carcinomas. In *Diagnostic Histopathology of the Breast*, Page DL and Anderson TJ. (eds), pp. 300–311. Churchill Livingstone: Edinburgh

Fishel RA, Lescoe MK, Rao MRS, Copeland NG, Jenkins NA, Garber J, Kane M and Kolodner R (1993) The human mutator gene homolog MSH2 and its association with hereditary nonpolyposis colon cancer. *Cell* **75**: 1027–1038

Fox SB, Leek RD, Weekes MP, Whitehouse RM, Gatter KC and Harris AL (1995) Quantitation and prognostic value of breast cancer angiogenesis: comparison of microvessel density, Chalkley count, and computer image analysis. *J Pathol* **177**: 275–283

Frame MC, Wilkie NM, Darling AJ, Chudleigh A, Pintzas A, Lang JC and Gillespie DAF (1991) Regulation of AP-1/DNA complex formation in vivo. *Oncogene* **6**: 205–209

Guedson J-L, Ternynck T and Avrameas S (1979) The use of avidin–biotin interaction in immunoenzymatic techniques. *J Histochem Cytochem* **27**: 1131–1139

Horak ER, Leek R, Klenk N, Lejeune S, Smith K, Stuart N, Greenall M, Stepniewska K and Harris AL (1992) Angiogenesis, assessed by platelet/endothelial cell adhesion molecule antibodies, as indicator of node metastasis and survival in breast cancer. *Lancet* **340**: 1120–1124

Hutchinson F (1985) Chemical changes induced in DNA by ionizing radiation. *Prog Nucleic Acid Res Mol Biol* **32**: 115–154

Ishibashi T, Nakabeppu Y, Kawate H, Sakumi K, Kayakawa H and Sekiguchi M (1995) Intracellular location and function of DNA repair methyltransferase in human cells. *Mutat Res* **315**: 199–212

Kakolyris S, Kaklamanis L, Engels K, Turley H, Hickson ID, Gatter KC and Harris AL (1997) Human apurinic endonuclease 1 expression in a colorectal adenoma–carcinoma sequence. *Cancer Res* **57**: 1794–1797

Kaplan EL and Meier P (1958) Non-parametric estimation from incomplete observations. *J Am Stat Assoc* **53**: 457–481

Leach FS, Nicolaides NC, Papadopoulos N, Liu B, Jen J, Parsons R, Peltomaki P, Sistonen P, Aaltonen LA, Nytromlahti M et al (1993) Mutations of a mutS homolog in hereditary nonpolyposis colorectal cancer. *Cell* **75**: 1215–1235

Lee SM, Harris M, Rennison J, McGown A, Bromley M, Elder RH, Rafferty JA, Crowther D and Marqison GP (1993) Expression of $O^6$-alkylguanine-DNA-alkyltransferase in situ, in ovarian and Hodgkin's tumors. *Eur J Cancer* **29A**: 1306–1312

Loeb LA and Preston BD (1986) Mutagenesis by apurinic/apyrimidinic sites. *Annu Rev Genet* **20**: 201–230

Malins DC, Holmes EH, Polissar NL and Gunselman SJ (1993) The etiology of breast cancer. Characteristic alteration in hydroxyl radical-induced DNA base lesions during oncogenesis with potential for evaluating incidence risk. *Cancer* **71**: 3036–3043

Needham GK, Nicholson S, Angus B, Farndon JR and Harris AL (1988) Relationship of membrane-bound tissue type and urokinase type plasminogen activators in human breast cancers to estrogen and epidermal growth factor receptors. *Cancer Res* **48**: 6603–6607

Robson CN and Hickson ID (1991) Isolation of cDNA clones encoding a human apurinic/apyrimidinic endonuclease that corrects DNA repair and mutagenesis defects in *E. coli* xth (exonuclease) mutants. *Nucleic Acids Res* **19**: 5519–5523

Robson CN, Milne AM, Pappin DJ and Hickson ID (1991) Isolation of cDNA clones encoding an enzyme from bovine cells that repairs oxidative DNA damage in vitro: homology with bacterial repair enzymes. *Nucleic Acids Res* **19**: 1087–1092

Seki S, Akiyama K, Watanabe S, Hatsushika M, Ikeda S and Tsutsui K (1991) cDNA and deduced amino acid sequence of a mouse DNA repair enzyme (APEX nuclease) with significant homology to *Escherichia coli* exonuclease III. *J Biol Chem* **266**: 20797–20802

Teoule R (1987) Radiation-induced DNA damage and its repair. *Int J Radiat Biol Relat Stud Phys Chem Med* **51**: 573–589

Thorgeirsson SS (1993) Endogenous DNA damage and breast cancer (editorial). *Cancer* **71**: 2897–2899

Tomkinson AE, Bonk RT and Linn S (1988) Mitochondrial endonuclease activities specific for apurinic/apyrimidinic sites in DNA from mouse cells. *J Biol Chem* **263**: 12532–12537

Walker LJ, Robson CN, Black E, Gillespie D and Hickson ID (1993) Identification of residues in the human DNA repair enzyme HAP1 (Ref-1) that are essential for redox regulation of jun DNA binding. *Mol Cell Biol* **13**: 5370–5376

Walker LJ, Craig RB, Harris AL and Hickson ID (1994) A role for the human DNA repair enzyme HAP1 in cellular protection against DNA damaging agents and hypoxic stress. *Nucleic Acids Res* **22**: 4884–4889

Wilson DM, Deutsch WA and Kelley MR (1994) Drosophila ribosomal protein S3 contains an activity that cleaves DNA at apurinic/apyrimidinic sites. *J Biol Chem* **269**: 25359–25364

Xanthoudakis S and Curran T (1992) Identification and characterization of Ref-1, a nuclear protein that facilitates AP-1 DNA-binding activity. *Embo J* **11**: 653–665

Xanthoudakis S, Miao G, Wang F, Pan YC and Curran T (1992) Redox activation of Fos-Jun DNA binding activity is mediated by a DNA repair enzyme. *Embo J* **11**: 3323–3335

Xanthoudakis S, Miao GG and Curran T (1994) The redox and DNA-repair activities of Ref-1 are encoded by nonoverlaping domains. *Proc Natl Acad Sci USA* **91**: 23–27

Yao KS, Xanthoudakis S, Curran T and O'Dwyer PJ (1994) Activation of AP-1 and of a nuclear redox factor, Ref-1, in the response of HT29 colon cancer cells to hypoxia. *Mol Cell Biol* **14**: 5997–6003

© Cancer Research Campaign 1998